# Characterization of antiseizure medications effects on the EEG neurodynamic by fractal dimension

**DOI:** 10.3389/fnins.2024.1401068

**Published:** 2024-06-07

**Authors:** Camillo Porcaro, Dario Seppi, Giovanni Pellegrino, Filippo Dainese, Benedetta Kassabian, Luciano Pellegrino, Gianluigi De Nardi, Alberto Grego, Maurizio Corbetta, Florinda Ferreri

**Affiliations:** ^1^Department of Neuroscience and Padova Neuroscience Center (PNC), University of Padova, Padova, Italy; ^2^Institute of Cognitive Sciences and Technologies (ISTC) – National Research Council (CNR), Rome, Italy; ^3^Centre for Human Brain Health, School of Psychology, University of Birmingham, Birmingham, United Kingdom; ^4^Neurology Clinics, Azienda Ospedale Università, Padua, Italy; ^5^Unit of Clinical Neurophysiology, Azienda Ospedale Università, Padua, Italy; ^6^Epilepsy Program, Schulich School of Medicine and Dentistry, Western University, London, ON, Canada; ^7^Veneto Institute of Molecular Medicine (VIMM), Fondazione Biomedica, Padua, Italy

**Keywords:** quantitative-EEG, focal epilepsy, antiseizure medications, biomarkers, fractal dimension

## Abstract

**Objectives:**

An important challenge in epilepsy is to define biomarkers of response to treatment. Many electroencephalography (EEG) methods and indices have been developed mainly using linear methods, e.g., spectral power and individual alpha frequency peak (IAF). However, brain activity is complex and non-linear, hence there is a need to explore EEG neurodynamics using nonlinear approaches. Here, we use the Fractal Dimension (FD), a measure of whole brain signal complexity, to measure the response to anti-seizure therapy in patients with Focal Epilepsy (FE) and compare it with linear methods.

**Materials:**

Twenty-five drug-responder (DR) patients with focal epilepsy were studied before (t1, named DR-t1) and after (t2, named DR-t2) the introduction of the anti-seizure medications (ASMs). DR-t1 and DR-t2 EEG results were compared against 40 age-matched healthy controls (HC).

**Methods:**

EEG data were investigated from two different angles: frequency domain—spectral properties in δ, θ, α, β, and γ bands and the IAF peak, and time-domain—FD as a signature of the nonlinear complexity of the EEG signals. Those features were compared among the three groups.

**Results:**

The δ power differed between DR patients pre and post-ASM and HC (DR-t1 vs. HC, *p* < 0.01 and DR-t2 vs. HC, *p* < 0.01). The θ power differed between DR-t1 and DR-t2 (*p* = 0.015) and between DR-t1 and HC (*p* = 0.01). The α power, similar to the δ, differed between DR patients pre and post-ASM and HC (DR-t1 vs. HC, *p* < 0.01 and DR-t2 vs. HC, *p* < 0.01). The IAF value was lower for DR-t1 than DR-t2 (*p* = 0.048) and HC (*p* = 0.042). The FD value was lower in DR-t1 than in DR-t2 (*p* = 0.015) and HC (*p* = 0.011). Finally, Bayes Factor analysis showed that FD was 195 times more likely to separate DR-t1 from DR-t2 than IAF and 231 times than θ.

**Discussion:**

FD measured in baseline EEG signals is a non-linear brain measure of complexity more sensitive than EEG power or IAF in detecting a response to ASMs. This likely reflects the non-oscillatory nature of neural activity, which FD better describes.

**Conclusion:**

Our work suggests that FD is a promising measure to monitor the response to ASMs in FE.

## 1 Introduction

Epilepsy represents one of the most common neurological conditions, affecting up to 1% of the world population. The clinical management of epilepsy patients is largely based on clinical judgment and is essentially qualitative. For instance, the choice of the first medication—after a diagnosis of epilepsy is made—is based on the clinical features of the seizures and the potential side effect profile of the medication. Similarly, the efficacy of response is established based on the reduction of the number of seizures or the qualitative improvement of inter-ictal EEG signals. Currently, there are no quantitative biomarkers for the prediction of pharmacological efficacy at the population or individual patient level.

The objective of this study is to quantitatively assess the effects of ASMs on brain signals to predict the likelihood of response through an automated algorithm leveraging quantitative analysis of clinical EEGs (qEEG) (Park et al., 2020). Quantitative EEG is a promising branch of clinical neurophysiology that explores local and global brain dynamics. When coupled with drugs (Jobert et al., 2012) qEEG is a promising tool to study the response to new drugs and has become an established technique for their classification (Galderisi et al., 1994; Mucci et al., 2006; Fink, 2010; Iosifescu, 2011).

So far, the two most reliable EEG biomarkers of ASM response are the interictal epileptiform discharges and the power spectral analysis, while the use of the Individual Alpha Frequency (IAF) peak is still debated but may represent a promising biomarker (Reynolds et al., 2023). However, these linear methods mainly capture the oscillatory component of the EEG signal and do not consider non-stationarities and non-linearities present in EEG signals (Stam, 2005; Klonowski, 2009). As stated by Cole and Voytek and by Jones and colleagues (Jones, 2016; Cole and Voytek, 2017), brain signals do not simply represent a sustained oscillation at a particular frequency but rather brief bouts of activity that repeat intermittently (Feingold et al., 2015; Lundqvist et al., 2016). Neuromodulation studies demonstrate that the application of complex and non-sinusoidal waveforms is more effective than sinusoidal oscillators in modulating brain (Somers and Kopell, 1993; Fröhlich and McCormick, 2010; Fröhlich, 2015; Dowsett and Herrmann, 2016; Cottone et al., 2018; Porcaro et al., 2019) and entraining brain rhythms (Somers and Kopell, 1993; Dowsett and Herrmann, 2016). This “hidden information” captured by non-linear methods such as fractal dimension analysis may be additional and complementary to linear methods and could shed light on the physiological neural communication, computation, and cognition in healthy as well as patients with neuropathological conditions (Goldberger, 2001; Goldberger et al., 2002; Zhang and Raichle, 2010; Rodríguez-Bermúdez and García-Laencina, 2015; Porcaro et al., 2017, 2019, 2020a,b, 2022). This is the reason why time-series fractal analysis is more and more used in different research fields ranging from basic neuroscience (Di Ieva et al., 2014, 2015; Moaveninejad et al., 2024), neurophysiology (Adeli et al., 2008; Ahmadlou and Adeli, 2012; Ahmadlou et al., 2012a,b), translational neuroscience (Smits et al., 2016; Porcaro et al., 2020b, 2021, 2022; Fiorenzato et al., 2024; Olejarczyk et al., 2024) to genetic variability in human phenotypes (Cattani and Pierro, 2013; Lee, 2020; Borri et al., 2022).

The purpose of our study is, therefore, to compare EEG signals in newly diagnosed patients with focal epilepsy patients before and after the initiation of therapy and examine their normalization using both linear (power spectra and IAF) and non-linear methods (FD). Specifically, we are interested in evaluating which method is more sensitive in detecting differences pre- and post-therapy with ASMs.

## 2 Materials and methods

### 2.1 Patients and data collection

We retrospectively reviewed the data of 25 newly diagnosed focal epilepsy patients and a control group of 40 healthy subjects enrolled at the epilepsy clinic of the Neurophysiological Unit of the Padua University Hospital (see Table 1). Epilepsy patients fulfilling the following inclusion criteria were included: (i) focal epilepsy according to the International League Against Epilepsy diagnostic recommendations (Scheffer et al., 2017); (ii) > 16 years old; (iii) no previous ASMs therapy (drug-naïve patients); (iv) at least two routine EEGs performed before (i.e., <30 days – DR-t1) and 6–12 months after (DR-t2) the beginning of treatment; (vi) EEGs included 5+ min of artifact free wakefulness; (vii) clinical follow-up at two-years. The exclusion criteria were: (i) other drugs acting on the CNS; (ii) medication change between EEG recordings. All patients underwent neurophysiological assessment, EEG, and brain MRI, as per standard of care (Koutroumanidis et al., 2017a,b; Scheffer et al., 2017).

**Table 1:** Demographic characteristics of the sample by group.

Group	N.	Age (years)		Gender		Etiology		Drug		
		Mean	SD	F	M	U	S	LEV	LTG	LCM
EPI	25	39.8	17.0	18	7	18	7	15	5	5
HC	40	38.9	16.0	23	17					

EPI, focal epilepsy patients; HC, healthy controls; N, number of individuals; SD, standard deviation; U, Unknown origin; S, Structural origin; LEV, Levetiracetam; LTG, Lamotrigine; LCM, Lacosamide.

Healthy subjects were volunteers. They were interviewed by a neurologist to rule out medical conditions potentially biasing the study. Healthy subjects met the following inclusion criteria: (i) age > 16 years; (ii) no medical or psychiatric conditions; (iii) no neuroactive drugs.

The study protocol was approved by Padua University Hospital’s ethics committee for a retrospective study.

### 2.2 EEG recording

Nineteen channel EEG was acquired with a EB-Neuro Galileo (Mizar 40) recorder. The electrodes were placed according to the international 10–20 system (Fp1, Fp2, F3, F4, C3, C4, P3, P4, F7, F8, T3, T4, T5, T6, O1, O2, Fz, Cz, Pz). The reference was placed on FPz and the ground on FCz. Impedance was kept below 10 kOhm for all electrodes. The sampling rate was set to 256 Hz. EEG recordings for patients lasted at least 20 min as per standard of care. This included 5 min of EEG eyes closed, which was selected for the purpose of this study. The remaining 15 min included eyes open recording, reactivity to eye closure and opening, intermittent photic stimulation, and hyperventilation, as per common clinical practice. The healthy control group performed 5 min of EEG recordings with open eyes and 5 min of EEG recordings with closed eyes.

### 2.3 EEG pre-processing

Quantitative EEG analysis was performed using the EEGLab Toolbox for Matlab^1^ and in-home Matlab code. Offline data pre-processing included: (i) visual inspection for rejection of possible interictal and ictal epileptiform activity; (ii) DC removal; (iii) bandpass filter between 1 and 48 Hz (linear phase finite impulse response filter); (iv) EEG re-reference to average; (v) correction for pulse and eye blink artifacts using Independent Component Analysis (ICA) (Barbati et al., 2004; Porcaro et al., 2015). Visual identification of interictal and ictal abnormalities was performed by experienced neurophysiologists blind to the clinical data as well as 5 min of EEG eyes closed (FF, CL, FD).

### 2.4 Characterization of electrophysiological neural activity at rest

We considered signal properties in the frequency domain (PSD) and time domain. The PSD is the squared modulus of the continuous Fourier transform. It is particularly useful for studying brain oscillations on a time scale of minutes, typical of an individual’s “stable state” (Schomer and Lopes Da Silva, 2012). As for the time domain, the signal power of neuronal assemblies, as a function of frequency, displays a “power law” function (Ramon and Holmes, 2015), and the exponent of this function corresponds to its fractality. Thus, we used temporal Higuchi’s fractal dimension (FD) (Higuchi, 1988) as a signature of neural dynamics underlying brain functions.

### 2.5 EEG power spectrum

We calculated the PSD using the Welch procedure (256 time points duration, Hanning window, and 60% overlap). We then investigated the spectral properties of the EEG total power in the classical frequency bands, such as δ (1–3 Hz), θ (4–7 Hz), α (8–13 Hz), β (14–30 Hz), and γ (31–48 Hz) bands (Chatrian et al., 1974). In addition, IAF was defined as the exact frequency in the α range containing the maximum power. It was calculated using an automated peak-detection algorithm (function RestingIAF on EEGLab) (Corcoran et al., 2018).

### 2.6 Higuchi’s fractal dimension

FD (Higuchi, 1988) is a non-linear measure of waveform complexity applied in the time domain. Discretised functions or signals can be analyzed as a segment of data X(1), X(2), …, X(N), where *N* is the total number of samples. From the starting time sequence, a new self-similar time series $X_{m}^{k}$ can be calculated as Eq. 1:


(1) $$X_{m}^{k}:x\left(m\right),x\left(m+k\right),x\left(m+2k\right),\ldots ,x\left(m+\mathrm{int}\left(\frac{N-m}{k}\right)k\right)$$

for *m =* 1, 2, …, *k* where *m* is the initial time; *k* is the time interval, *k =* 1, 2, …, *k_max_*; *k_max_* is a free parameter, and *int()* represent the integer operator.

The length, *L_m_(k)*, of each curve *X^k^_m_* is calculated as Eq. 2:


(2) $$\begin{array}{l} L_{m}\left(k\right)=\\ \frac{1}{k}\left[\underset{i=1,\mathrm{int}\left(\frac{N-m}{k}\right)}{\sum }\left|X\left(m+ik\right)-X\left(m+\left(i-1\right)\right)k\right|\frac{N-1}{\mathrm{int}\left(\frac{N-m}{k}\right)k}\right] \end{array}$$

where *N* is the length of the original time series *X* and $\left(\frac{N-1}{\mathrm{int}\left(\frac{N-m}{k}\right)k}\right)$ is a normalization factor.

*L_m_(k)* was averaged across all m forming the mean value of the curve length *L(k)* for each *k =* 1,…, *k_max_* as Eq. 3:


(3) $$L\left(k\right)=\frac{\sum_{m=1}^{k}L_{m}\left(k\right)}{k}$$

An array of mean values *L(k)* was obtained and the FD was estimated as Eq. 4:


(4) $$FD=\log\left(L\left(k\right)\right)/\log\left(1/k\right)\;for\;k=1,2,\ldots ,k_{\text{max}}$$

In practice, the original curve or signal can be divided into smaller parts with or without overlap, called “windows.” Then, the method for computing the FD should be applied to each window where N should be seen as the length of the window. Individual FD values can be averaged across all windows for the entire curve (or data time-series), and the mean FD value can be used as a measure of curve complexity. Additional analysis demonstrated that FD measurements were not dependent on the choice of window length and overlapping windows see Smits et al. (2016), Marino et al. (2019), and Porcaro et al. (2020a) for details. Here, for each EEG channel, we calculated FD in non-overlapping time windows of 1 s (corresponding to 256-time points since our sample frequency rate was 256 Hz) as a good compromise between the window length of the data and computational time. The choice of the free parameter k has a crucial role in FD estimation; for this reason, for each window, we estimated 127 values of FD for all the possible *k* values (i.e., *k* = 2, …, 128).

The value 128 was equal to half of the samples within our 1 s window (i.e., 128-time points are the maximum that can be chosen since the maximum *k* value is equal to half of the window length). For the subsequent FD analysis, we set *k* = 25 (Smits et al., 2016; Marino et al., 2019; Porcaro et al., 2020a, 2022).

### 2.7 Statistical analysis

Shapiro–Wilk test for normality revealed that PSD, IAF, and FD values did not differ from a Gaussian distribution (*p* > 0.200). Repeated-measures analysis of variance (rm-ANOVA) was performed on PSD values to investigate the interaction effect GROUPs × BANDs (the three GROUPs as a between-subject factor: DR-t1, DR-t2, and HC); the five BANDs as a within-subjects factor (δ, θ, α, β, γ). The sphericity of the covariance matrix was verified with the Mauchly sphericity test. In the case of violation of the sphericity assumption, the Greenhouse–Geisser epsilon adjustment was used. One-way ANOVA was also applied to investigate the GROUPs effect (between-subject factor: DR-t1, DR-t2, and HC) on FD and IAF. The results were analyzed only for all ANOVA models if the Wilks’ Lambda multivariate significance criterion was achieved. In the case of violation of the sphericity assumption, the Greenhouse–Geisser epsilon adjustment was used. *Post-hoc* analysis was performed using the Bonferroni correction method for multiple comparisons. Finally, the three measures (PSD, IAF, and FD) were analyzed using a Bayesian approach to test which of the three methods was better able to discriminate between DR-t1 and DR-t2 conditions. We have performed the same ANOVA test as above on a single drug to test for any drug specificity. The drug chosen was LEV since it has higher numerosity, with the number of subjects being 15. All the analyses described above were conducted in JASP software (v0.17.2-1—jasp-stats.org/).

## 3 Results

### 3.1 Demographic results

A one-way ANOVA model found no significant age difference among groups [*F*(2, 89) = 2.24, *p* = 0.129].

### 3.2 EEG power spectrum and IAF

#### 3.2.1 All drugs (LEV, LTG, LCM)

**Figure 1:**
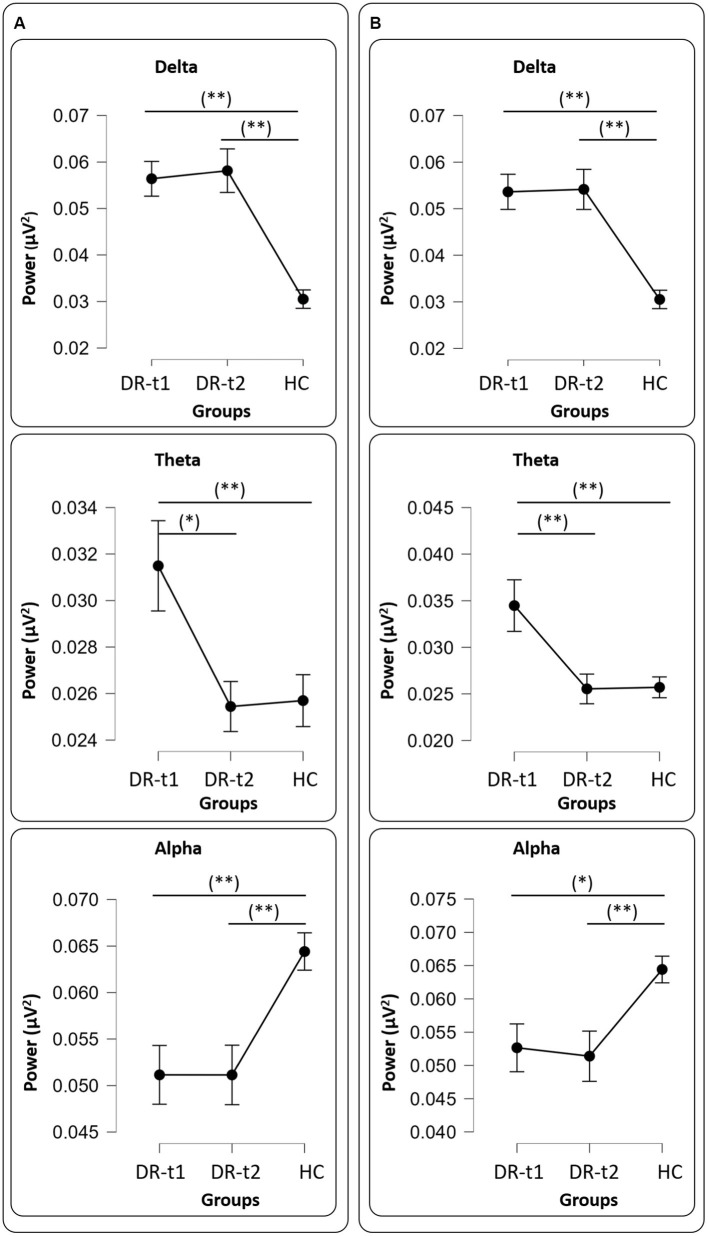
Panel **(A)** all drugs, Delta (Upper panel), Theta (Middle panel), and Alpha (Bottom panel) values among groups [healthy controls (HC), patients before pharmacological intervention (DR-t1), and patients after pharmacological intervention (DR-t2)]. The horizontal bar indicates which contrast reached the significant level at *p* < 0.01 (**) and *p* < 0.05 (*). Black points and error lines represent the mean and the standard error. Panel **(B)**, as panel **(A)**, but for only LEV drug.

**Figure 2:**
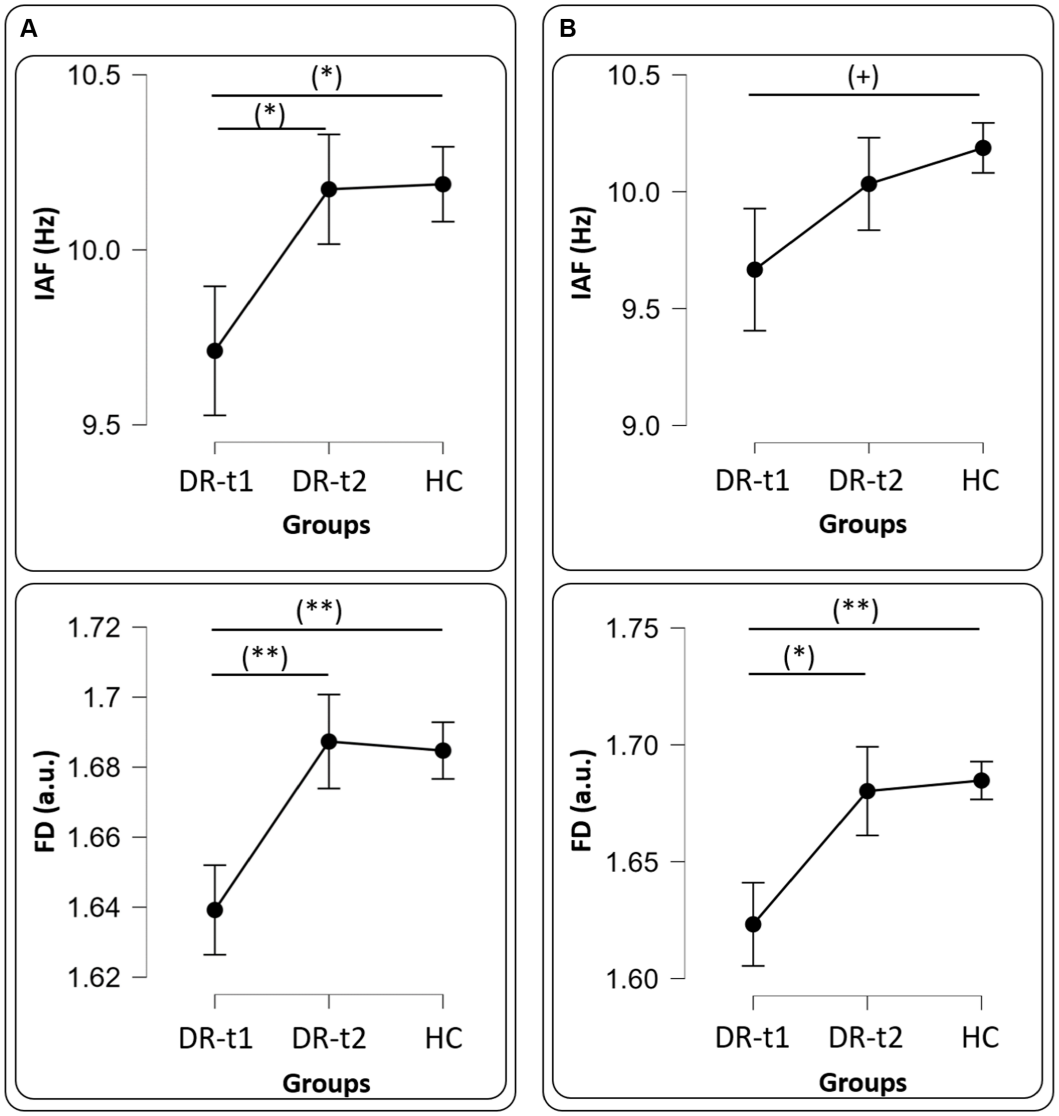
Panel **(A)** all drugs, IAF (Upper panel) and FD (Bottom panel) values among groups [healthy controls (HC), patients before pharmacological intervention (DR-t1), and patients after pharmacological intervention (DR-t2)]. The horizontal bar indicates which contrast reached the significant level at *p* < 0.01 (**), *p* < 0.05 (*) and (+) *p* = 0.09. Black points and error lines represent the mean and the standard error. Panel **(B)**, as panel **(A)**, but for only LEV drug.

A repeated measure ANOVA (rm-ANOVA) for the PSD with a Greenhouse–Geisser correction (Mauchly’s *W* = 0.005, *p* < 0.001, ε = 0.369) revealed a significant GROUP × BAND interaction [*F*(8, 356) = 16.4, *p* < 0.001]. The between-subjects factor GROUP also showed a difference [*F*(2, 89) = 26.1, *p* < 0.001]. *Post-hoc* tests using the Bonferroni correction revealed that the δ band was different between DR-t1 and HC (*p* < 0.001) and between DR-t2 and HC (*p* < 0.001). There was no significant difference between DR-t1 and DR-t2. The θ band differed between DR-t1 and DR-t2 (*p* = 0.015) and DR-t1 and HC (*p* = 0.01), but not between DR-t2 and HC. Finally, the α band significantly differed between DR-t1 and HC and between DR-t2 and HC (*p* = 0.002 for both), but there was no difference between DR-t1 and DR-t2. No significant differences were found for other frequency bands (Figure 1A). The IAF ANOVA model revealed a statistically significant difference between GROUPs [*F* (2, 89) = 3.29, *p* = 0.042]. Bonferroni corrected *post-hoc* tests revealed that the IAF value was lower for the DR-t1 as compared to the DR-t2 (*p* < 0.048) and the HC group (*p* = 0.042). No difference was observed for the DR-t2 vs. HC (Figure 2A, Up).

#### 3.2.2 Only LEV

rm-ANOVA for the PSD with a Greenhouse–Geisser correction (Mauchly’s *W* = 0.0113, *p* < 0.001, ε = 0.410) revealed a significant GROUP × BAND interaction [*F*(8, 268) = 14.2, *p* < 0.001]. The between-subjects factor GROUP also showed a difference [*F*(2, 67) = 18.9, *p* < 0.001]. *Post-hoc* tests using the Bonferroni correction revealed that the δ band was different between DR-t1 and HC (*p* < 0.001) and between DR-t2 and HC (*p* < 0.001). There was no significant difference between DR-t1 and DR-t2. The θ band differed between DR-t1 and DR-t2 (*p* = 0.008) and DR-t1 and HC (*p* = 0.001), but not between DR-t2 and HC. Finally, the α band significantly differed between DR-t1 and HC (*p* = 0.015) and between DR-t2 and HC (*p* = 0.006), but there was no difference between DR-t1 and DR-t2. No significant differences were found for other frequency bands (Figure 1B). The IAF ANOVA model only showed a tendency to significantly differ between GROUPs [*F* (2, 67) = 2.455, *p* = 0.09; Figure 2B, Up].

### 3.3 Higuchi’s fractal dimension

#### 3.3.1 All drugs (LEV, LTG, LCM)

The ANOVA model for the FD feature also revealed a significant GROUP effect for FD [*F*(2, 89) = 5.537, *p* = 0.005]. Bonferroni corrected *post-hoc* tests revealed that FD was lower for DR-t1 compared to DR-t2 (*p* = 0.015) and the HC group (*p* = 0.011). No significant difference was found for DR-t2 vs. HC (Figure 2A, Bottom).

#### 3.3.2 Only LEV

The ANOVA model for the FD estimated only on the LEV drug revealed a significant GROUP effect for FD [*F*(2, 67) = 5.903, *p* = 0.004]. Bonferroni corrected *post-hoc* tests revealed that FD was lower for DR-t1 compared to DR-t2 (*p* = 0.024) and the HC group (*p* = 0.004). No significant difference was found for DR-t2 vs. HC (Figure 2B, Bottom).

### 3.4 Is FD more sensitive than PSD or IAF in detecting drug response effects?

We used Bayesian Paired samples *t*-tests to compare the relative sensitivity of FD vs. PSD in discriminating DR-t1 vs. DR-t2 patients. There are several advantages to using Bayesian methods. First, Bayesian methods allow inferences about both the null and alternative hypotheses. Second, it is possible to compare Bayes Factors (BF) across analyses and, based on the magnitude of the BF, derive whether one result is more robust than another.

**Figure 3:**
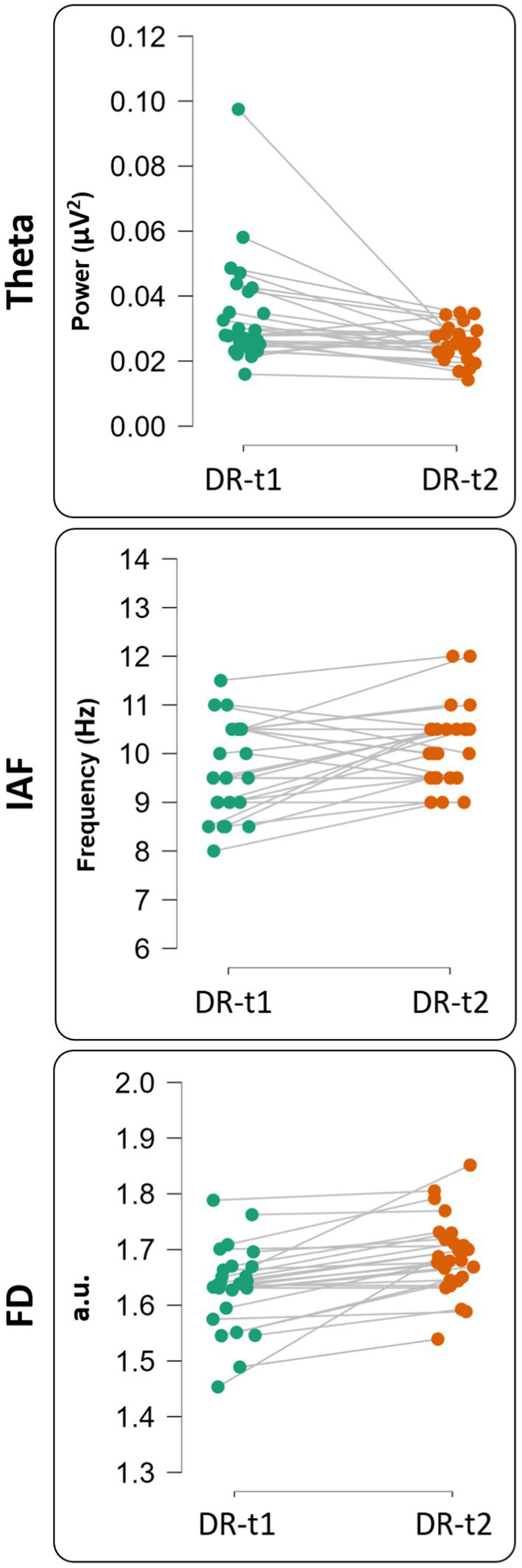
Single subject representation for theta value [upper panel—before (DR-t1) and after (DR-t2) pharmacological intervention], IAF value [middle panel—before (DR-t1) and after (DR-t2) pharmacological intervention] and FD value [bottom panel—before (DR-t1) and after (DR-t2) pharmacological intervention]. Each circle represents a subject, and the gray line connects the same subject before (green circle) and after (orange circle) pharmacological intervention.

**Figure 4:**
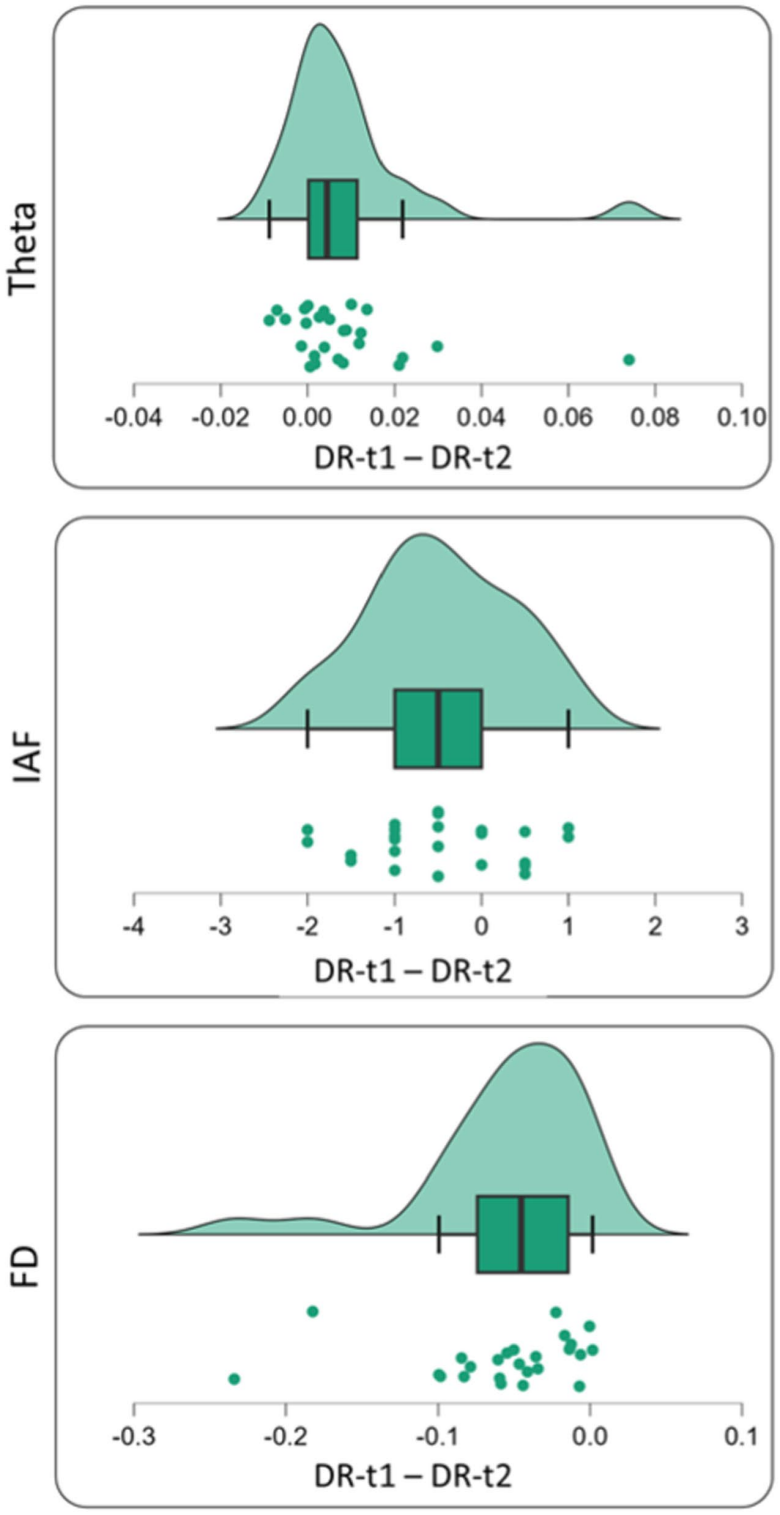
Single subject difference distribution density for theta value [Upper panel—before (DR-t1) and after (DR-t2) pharmacological intervention], IAF value [middle panel—before (DR-t1) and after (DR-t2) pharmacological intervention] and FD value [bottom panel—before (DR-t1) and after (DR-t2) pharmacological intervention]. Each green circle represents the subject difference for each feature under investigation (Theta Band, IAF, and FD) before and after pharmacological intervention. The green area under the curve represents its density distribution. In the box plot, the bold black line shows the sample median. The hinges indicate the 25th and 75th quantiles and the whiskers point to 1.5 interquartile ranges beyond the hinges.

We found a significant difference between DR-t1 and DR-t2 for FD [Student’s *t* (25) = −5.172, *p* < 0.001, BF_10_ = 973.069], θ band power [Student’s *t* (25) = 2.730, *p* = 0.011, BF10 = 4.220], and IAF [Student’s *t* (25) = −2.816, *p* = 0.009, BF_10_ = 4.994]. However, the magnitude of BF was higher for FD than θ band or IAF (BF FD = 973.069; BF θ band = 4.994; BF IAF = 4.220). Accordingly, FD was 195 times more likely than IAF, and 231 times more likely than θ band to distinguish DR-t1 from DR-t2. Figures 3, 4 show the descriptive statistics. In particular, Figure 3 shows the value for the θ band, IAF, and FD and each patient before and after the pharmacological intervention; the gray line that conjuncts the green circle with the orange circle emphasizes the trend obtained for each patient. The more the trend follows the same direction (i.e., from higher to lower θ), the higher will be the statistical effects. Figure 4 displays the individual cases (green dots), box plots, and density for the difference between the measures. In our case, FD (Figure 4, Bottom panel) clearly shows that the pharmacological intervention decreases the brain complexity estimated by Higuchi’s FD in all patients.

## 4 Discussion

The issue of assessing the response of ASMs in newly diagnosed epilepsy patients is an important clinical problem with important implications for health national systems. Research in the last 40 years has been devoted to developing EEG biomarkers as reliable indices of favorable response (Porcaro et al., 2019, 2020a, 2022; Comanducci et al., 2020). Compared to MRI and PET scans, EEG has many advantages, including low costs, widespread availability in economically less developed countries, non-invasiveness, and portability. However, EEG biomarkers are not routinely used in clinical practice to assess response. Patients are still evaluated based on clinical response and a qualitative evaluation of EEG recordings and interictal abnormalities.

The research area exploring the relationship between fractal dimension and epilepsy is a novel and promising field, delving into the dynamics of brain activity. The Fractal dimension is a measure of the irregularity or complexity of a geometric structure, such as the spatiotemporal dynamics of brain (Porcaro et al., 2024). In epilepsy, the pathological mechanism involves abnormal synchronization of neuronal activity, leading to seizures. Fractal EEG signal analysis might be a new tool to investigate this abnormal complexity of brain activity in epileptic patients (Jouny and Bergey, 2012; Khoa et al., 2012).

In healthy individuals, EEG signals exhibit a certain level of complexity, characterized by a fractal dimension within a specific range (Cottone et al., 2017; Marino et al., 2019). However, in epileptic patients, this complexity may be altered due to disruptions in the normal functioning of neural networks (Jouny and Bergey, 2012; Khoa et al., 2012). Several studies have found differences in fractal dimension measures between healthy individuals and epileptic patients, as well as between different types of epilepsy. For example, some research suggests that the fractal dimension of EEG signals may decrease during epileptic seizures, indicating a loss of complexity in brain activity (Olejarczyk, 2003; Jouny and Bergey, 2012; Khoa et al., 2012). On this basis, fractal analysis has an enormous potential for understanding epilepsy, with implications for both diagnosis and treatment. By providing insights into the underlying mechanisms of epilepsy, this analysis could pave the way for developing more effective diagnostic tools and therapies, making it a crucial area of study.

Overall, while the exact connection between the fractal dimension and the pathological mechanism of epilepsy is still being explored, research in this area holds promise for advancing our understanding of this complex neurological disorder.

With this aim, here we used Higuchi’s Fractal Dimension (FD) to investigate newly diagnosed patients with focal epilepsy from healthy controls and their response to ASMs. As a benchmark, we compared the FD to linear neurophysiological markers like the band-limited power and the IAF that have been evaluated in previous studies of response to ASMs (Clemens et al., 2007; Cho et al., 2012; Guo et al., 2014).

We found that ASMs treatment reduced θ power near normal levels, while α and δ power bands did not change pre- and post-treatment (Figure 1). ASMs also normalized the IAF and FD near the level of HC subjects (Figure 2).

Our reduction of θ band power after treatment with ASMs is generally consistent with several previous studies, and it does not appear to be drug-dependent. Patients treated with Levetiracetam showed an increase in α e β power and a decrease in δ and θ power bands (Cho et al., 2012). Patients treated with Perampanel showed an increase in α power in drug responder patients (Lanzone et al., 2021). In TLE patients, Levetiracetam induced an increase in α power and a decrease in θ band power (Ricci et al., 2021). In another study, patients treated with LTG or VPA as first line therapy showed reduced θ and γ power (Clemens et al., 2007; Clemens, 2008; Guo et al., 2014). However, other studies have shown spontaneous longitudinal fluctuations in power which may confound some of the drug response effect (Viana et al., 2021).

While in our study the α power did not significantly change pre- post-treatment, the θ power normalized consistently with previous studies. In addition, we showed an increase in IAF that normalized post-therapy. The IAF is a stable index of oscillatory activity in the occipital lobe that grows in the course of development, and it is decreased in some pathological conditions (e.g., schizophrenia, Ramsay et al., 2021). A within-subject study showed that IAF was significantly higher during a demanding working memory task than during rest or passive visual stimulation (Haegens et al., 2014). Our interpretation is that the increase in IAF reflects an improvement toward normalization of cognitive processing in our patients after starting ASMs.

Overall, our results are consistent with those of other studies that have used classical FFT-based linear methods to distinguish between healthy subjects and epileptic patients and between pre- and post-ASMs (Clemens et al., 2014; Pellegrino et al., 2018; Lanzone et al., 2021).

The most novel aspect of our results is that the FD increased normalizing after ASMs. Moreover, the FD was more sensitive than θ power and IAF in separating epileptic patients before and after treatment. The Fractal Dimension has been introduced as a marker in both healthy (Cottone et al., 2017; Marino et al., 2019; Porcaro et al., 2020a) and pathological conditions (Smits et al., 2016; Porcaro et al., 2019, 2020b, 2021, 2022). In the case of EEG, FD is a non-linear measure of signal complexity. Increased EEG synchrony results in its reduction while EEG desynchronization leads to FD increases. FD increases with task complexity (Cottone et al., 2018), and it is reduced after brain stroke (Zappasodi et al., 2014). The sensitivity of FD comes from its ability to estimate both oscillatory and non-oscillatory components of the EEG signal capturing patterns of activity that are not consistent over time. These non-rhythmic patterns have been clearly demonstrated in the human motor cortex (Feingold et al., 2015; Lundqvist et al., 2016; Cole et al., 2017; Cole and Voytek, 2017). The non-rhythmic nature of brain activity has been further supported by neuromodulation studies in which non-sinusoidal patterns were more effective in entraining brain rhythms (Somers and Kopell, 1993; Fröhlich and McCormick, 2010; Dowsett and Herrmann, 2016; Cottone et al., 2018).

Our results suggest that ASMs normalized brain activity in our focal epilepsy patients specifically by increasing signal complexity as indexed by FD. The corollary increase in IAF that has been associated with higher cognitive processing is also another neurophysiological indicator of brain activity normalization. It would have been important to show an improvement in neuropsychological scores post-therapy. It is remarkable that these changes occurred in the absence of apparent seizure activity. The large superiority of FD over oscillatory biomarkers (195 times more likely than IAF; 231 times higher than θ band) in picking up patients post-therapy is an indication that under physiological conditions brain activity is not oscillatory. To our knowledge, this is the first report to show a decrease of brain signal complexity in newly diagnosed epileptic subjects, which normalized after therapy.

## 5 Limitations and conclusions

The study suffers from several limitations. The sample is small, retrospective, and heterogeneous in terms of number of etiologies and drugs employed. In this respect, we have performed the ANOVA on a more heterogeneous subgroup concerning the drug (in particular, we selected the 15 subjects treated with the LEV). The ANOVA showed comparable results with respect to the results obtained with the entire group treated with different drugs (i.e., 15 LEV, 5 LTG, and 5 LCM). The robustness of our findings despite these limits may suggest that the effect is robust and even stronger in a more homogenous sample. Another limitation is that we cannot exclude that these effects underlie the normal longitudinal recovery of signal complexity after the occurrence of novel seizure activity. To rule out this possibility, it would be important to have a group of non-responders and follow them longitudinally. However, non-responders are only about 30% of all epileptic patients, and a comparison across groups would be less sensitive than a within-subject comparison as in our study. The mean change in IAF pre- post-therapy was, on average ~ 0.5 Hz which is on par with within-subject IAF variability (~0.9 Hz in Haegens et al.). In contrast, IAF between-subject variability is much larger (2.9 Hz in Haegens), which would require a much larger sample of both responders and non-responders given the effect size observed here.

In conclusion, we propose a new quantitative and automatic measure to track response to therapy in focal epilepsy. Future prospective studies are needed to validate this finding.

## Data availability statement

The data that support the findings of this study are available on request from the corresponding author. The data are not publicly available due to privacy or ethical restrictions.

## Ethics statement

The studies involving humans were approved by Padua University Hospital’s ethics committee. The studies were conducted in accordance with the local legislation and institutional requirements. The participants provided their written informed consent to participate in this study.

## Author contributions

CP: Conceptualization, Data curation, Formal analysis, Methodology, Supervision, Writing – original draft, Writing – review & editing. DS: Writing – original draft, Writing – review & editing. GP: Writing – review & editing. FD: Writing – review & editing. BK: Writing – review & editing. LP: Writing – review & editing. GD: Writing – review & editing. AG: Writing – review & editing. MC: Conceptualization, Supervision, Writing – original draft, Writing – review & editing. FF: Conceptualization, Data curation, Supervision, Writing – original draft, Writing – review & editing.

## Footnotes

^1^ https://sccn.ucsd.edu/eeglab/index.php
